# Structure and catalytic behaviour of CuO–CeO_2_ prepared by high-energy ball milling

**DOI:** 10.1098/rsos.181861

**Published:** 2019-02-06

**Authors:** Nguyen The Luong, Hideyuki Okumura, Eiji Yamasue, Keiichi N. Ishihara

**Affiliations:** Department of Socio-Environmental Energy Science, Graduate School of Energy Science, Kyoto University, Yoshida Honmachi, Sakyo-ku, Kyoto 606-8501, Japan

**Keywords:** CeO_2_, CuO, ZrO_2_, H_2_-temperature-programmed reduction, oxygen storage capacity

## Abstract

The aim of this study is to prepare CuO–CeO_2_ composite by means of mechanical milling and to investigate its characteristics as a catalyst. The structural and morphological features of milled samples are observed by X-ray diffractometry and scanning electron microscopy. The redox property and total OSC (oxygen storage capacity) of the milled sample were measured by using GC-TCD and TG-DTA, which are important parameters to indicate the effectiveness of catalysts. Interestingly, reduction of CuO is repeatedly observed when milling of CuO–CeO_2_ powder mixture is processed in air. The redox property of milled CuO–CeO_2_ sample is investigated by H_2_-TPR, where three reduction peaks are observed for 0 h milling and only one broad peak for various other milling times. The total OSC of mechanically driven CuO–CeO_2_ catalyst is much higher than that of the CeO_2_–ZrO_2_ traditional catalyst system at low temperatures.

## 1. Introduction

Nowadays, more than 95% of vehicles produced are equipped with a catalytic converter [1]. The three-way catalysts (TWCs), used for the gasoline-fuelled engine are capable of simultaneously converting CO, hydrocarbon (HC) and NO*_x_*, with a stoichiometric air-to-fuel ratio (A/F = 14.7), into harmless CO_2_, H_2_O and N_2_ [1]. Oxygen storage capacity (OSC) is one of the crucial factors for the performance of TWCs. The CeO_2_–ZrO_2_ composite is well known as an excellent promoter of OSC, where CeO_2_ exhibits the oxygen storage/release behaviour by redox variation of Ce ions between Ce^3+^ and Ce^4+^, while the introduction of ZrO_2_ into CeO_2_ improves the reduction temperature of ceria through structural modification of the ceria lattice [2], although the OSCs at low temperatures are still not high [1]. Many studies on CeO_2_-based materials have been reported, such as CeO_2_–Al_2_O_3_ [3], CeO_2_–SiO_2_ [4], CeO_2_–La_2_O_3_ [3,5,6], CeO_2_–TbO*_x_* [7], CeO_2_–PrO*_x_* [8] and CeO_2_–MO*_x_* (M: Zr, Ti, Cu) [9,10], to improve the OSC and increase the thermal stability.

As legislation becomes tighter, it is necessary to improve the efficiency of TWCs at low temperatures under an oxygen-rich atmosphere. The copper/copper oxides are known to exhibit oxygen storage/release behaviour at low temperatures, although it causes fragmentation due to the large volume change [11]. Then, various metal oxides without large volume change, such as CuMO_2_ (M = Al, Fe, Mn, Ga), have been investigated to reduce fragmentation [11], where the reduction of Cu^2+^ to Cu is studied by the H_2_-TPR (temperature-programmed reduction) and the OSC is improved at lower temperatures.

It is known that CuO–CeO_2_ mixed oxides exhibit high levels of oxidation of carbon monoxide and hydrocarbon [12–15], SO_2_ reduction by CO [16–18], NO reduction [19] and phenol oxidation [20–22]. It has also been shown that the redox properties and catalytic performance strongly depend on the preparation methods, such as sol–gel [23], hydrothermal routes [24,25], precipitation method [26], reverse micelle [27], sonochemical [28], chemical vapour deposition [29], flux method [30], micro-wave heating [31] and surfactant-assisted method [32]. The preparation condition and the mixed-oxide composition influence the phase, morphology and distribution of copper species on ceria. The enhanced catalytic activity results from interactions among the copper–cerium oxide phases.

Mechanical milling has long been used to prepare non-equilibrium materials, solid solutions and other metastable phases and also to drive mechanochemical reactions. It has been shown that, because the enhanced reaction rate can be achieved and dynamically maintained during milling as a result of microstructural refinement and mixing processes accompanying repeated fracture, deformation and welding of particles during collision events [33], several treatments employing milling could be applied for various preparation stages of mixed oxides [34–37]. Recently, the mixed oxides containing CeO_2_ and other dopants, such as ZrO_2_, TbO*_x_* and HfO_2_, have been prepared by mechanical milling [7,38] with strong enhancement of the OSC properties of CeO_2_. In addition, Castricum *et al*. report that the milling process of mixed Cu, Cu_2_O or CuO and ZnO in synthetic air results in oxidation of Cu precursors, while, under vacuum, it results in reduction. They also report that the mechanochemical reactions are promoted by mechanical milling in the presence of ZnO [39].

The OSC property of the CuO–CeO_2_ system for TWCs has not been previously reported, and it is interesting to study the effect of the mechanochemical process on the OSC property of the Ce–Cu–O systems. It is also reasonable to consider that the valence change of Ce^4+^/Ce^3+^ and/or Cu^2+^/Cu^+^/Cu may improve the OSC property at lower temperatures. Thus, the primary aim of this study is to characterize the CeO_2_–CuO mixed oxides prepared by high-energy mechanical milling and evaluate the OSC properties, which is compared with CeO_2_–ZrO_2_ traditional catalysts prepared under the same experimental conditions.

## 2. Experiment

### 2.1. Catalyst preparation

Monoclinic CuO (Nilaco Corporation, less than 250 µm, 99.999% purity), monoclinic ZrO_2_ (Nilaco Corporation, less than 200 µm, 99.8% purity) and cubic CeO_2_ (Kojundo Chemical, less than 180 µm, 99.99% purity) powders were used as starting materials. The molar ratio of CuO in the composite was changed to be 0, 20, 30, 50, 80 and 100%. High-energy vibratory ball milling (Super-Misuni, Nissin Giken Co. Ltd.) was employed, with a rotational speed of 710 r.p.m., where the milling atmosphere was ambient. The powders and zirconia balls (ϕ10 mm) were charged in a stainless steel vial (ϕ100 mm), where the ball-to-powder weight ratio was 18 : 1 (18 g balls per 1 g powder) and the milling durations were changed from 0 to 30 h.

### 2.2. Characterizations

The structures, the morphological aspects and the compositions of milled samples were analysed by X-ray diffractometry (XRD) using Cu K*α* radiation (RIGAKU RINT-2100CMT), scanning electron microscopy (SEM, JEOL, JSM-5800) and EDX (energy-dispersive X-ray). The silver powder (99.8% purity) was used for both the 2-theta calibration of X-ray diffraction line positions and the background intensity calibration, and the lattice parameter and the crystallite size were then calculated on the basis of FWHM (full width at half maximum intensity) of the 220 peak of CeO_2_, where the Scherrer's Equation was used for the latter. The surface areas were estimated by the N_2_ adsorption method (single-point BET).

### 2.3. Catalytic property measurements

The milled sample was subjected to measurement of total OSC according to the method by Tanabe *et al.* [40] and Morikawa [41]. The weight change of the milled sample was measured with TG-DTA (RIGAKU TG-8120) by the following procedure; the milled sample (about 20 mg) on an alumina container was completely oxidized at 500°C in a N_2_-20vol%O_2_ mixed gas flow (dry air, 500 ml min^−1^) for 60 min, followed by cooling to 300°C. Then, the gas atmosphere was switched to an Ar-5vol%H_2_ flow (500 ml min^−1^) and the weight decrease due to reduction was monitored until no weight change was observed. Afterward, the gas atmosphere was again switched to the dry air and the weight increase due to oxidation was monitored until no weight change was observed. The procedure was repeated twice.

The dynamic reduction behaviour was measured by TPR, where the milled sample (about 50 mg) was put in a quartz reactor and heated at 400°C for 1 h under a N_2_-20%O_2_ gas flow (30 ml min^−1^) and cooled to room temperature (RT). The gas was then changed to Ar-5%H_2_ (25 ml min^−1^) and the sample was heated at 15^o^C/min for the temperature range of 35–900°C, where the H_2_ consumption was measured by GC-TCD (Varian CP-4900). For the second run of H_2_-TPR, the sample (50 mg) after the first TPR was cooled to RT in Ar-5%H_2_ gas, re-oxidized at 400°C for 1 h under a N_2_-20%O_2_ gas flow (30 ml min^−1^), cooled to RT again, and finally heated under an Ar-5%H_2_ gas flow (25 ml min^−1^) at 15^o^C min^−1^ up to 900°C for the repeated H_2_-TPR.

## 3. Results and discussion

### 3.1. Structural characterization

**Figure 1.**
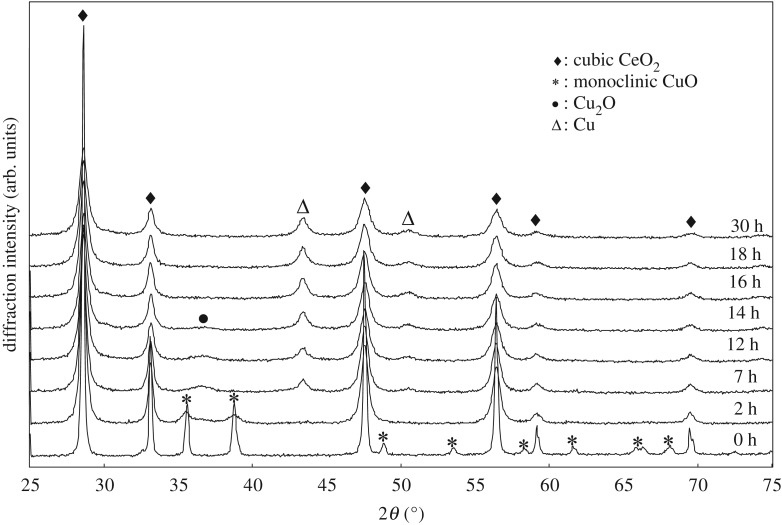
XRD patterns of (CuO)_0.5_(CeO_2_)_0.5_ powder (CuO: monoclinic, CeO_2_: cubic) with milling time.

The XRD patterns of milled samples, having a composition of 50%mol CuO and 50%mol CeO_2_, are shown in figure 1. The reflection peak intensities of the CuO phase are largely reduced with peak broadening after 2 h milling, while no appearance of other phases is detected. Although not detected, some CuO phases may exist in nanostructured or amorphous states. After 7 h milling, the CuO peaks are almost eliminated and replaced instead by the appearance of the faint reflections from a Cu_2_O phase and the clear peaks of fcc Cu. The coexistence of both phases lasts until the milling duration of around 14 h. The Cu_2_O reflections become weaker with milling, while the Cu peaks become more distinguished with milling, a tendency which continues up to 30 h milling. There is no change in the observed phase of CeO_2_ up to 30 h milling, although the peak broadening with milling is significant, particularly during the early milling stages regarding the change of the peak shape.

From the XRD results, the reductive valence change of CuO, i.e. Cu^2+^ → Cu^1+^ → Cu or Cu^2+^ → Cu, occurs during milling. This is consistent with a report that there are three ways [12,13] to reduce Cu^2+^ to Cu: (i) CuO → Cu_4_O_3_ → Cu_2_O → Cu, (ii) CuO → Cu_2_O → Cu or (iii) CuO → Cu. The presence of Cu is also confirmed by nuclear magnetic resonance spectroscopy in the 7 h- and 18 h-milled samples (not shown here). When the CeO_2_ and CuO powder phases are forced to contact at the bounding interphase interface during milling the cations of Ce^4+^ (or Ce^3+^ for non-stoichiometric sites especially near the surface) could be interchanged with Cu^2+^ cations through the vacancy mechanism. The CeO_2_ powder, in particular the surface volume, exhibits non-stoichiometric compositions with various defects [42], which can be formed upon the introduction of metal cations with higher or lower valences into CeO_2_. It is also known that the mechanical milling of powders induces the fracture and deformation through high-energy collisions among balls, vial surface and particles [43], leading to a modification of the crystal structure as well as large lattice strain. In this study, the Cu cations can be introduced in the lattice of CeO_2_, replacing the Ce cations as well as producing the extra oxygen vacancies in the CeO_2_ lattice.

**Figure 2.**
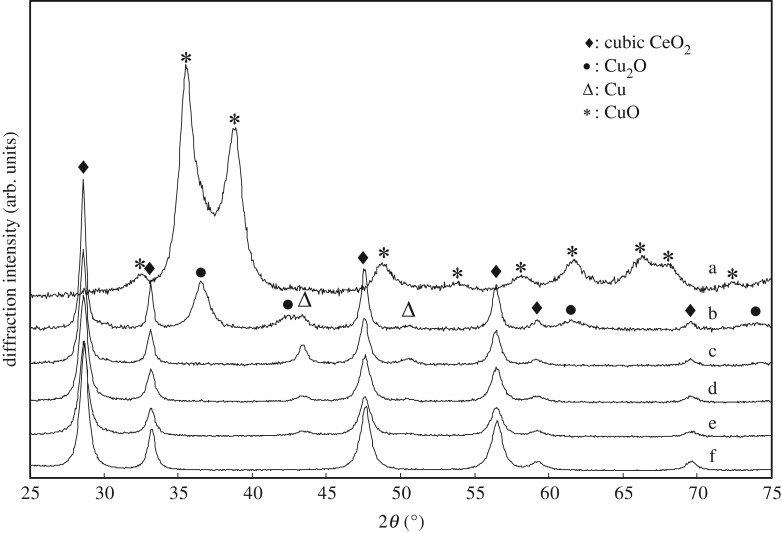
XRD patterns (CuO)_x_(CeO_2_)_1−x_ powder after 18 h milling (*x*=: (a) 1, (b) 0.8, (c) 0.7, (d) 0.5, (e) 0.2, (f) 0).

Figure 2 shows XRD patterns of 18 h-milled samples with various CeO_2_–CuO content ratios. For pure CuO (figure 2*a*), no phase change of CuO is observed after 18 h milling. But, with the 80 mol% CuO composite (figure 2*b*), the coexistence of both Cu and Cu_2_O phases is observed replacing the CuO, besides the existence of the cubic CeO_2_. With 70 mol% CuO (figure 2*c*), the observable Cu-related phase after 18 h milling is an fcc Cu phase only. By further reducing the CuO content (figure 2*d–f*) for each after 18 h milling, the emerged intensity of the Cu phase is gradually lowered, without a major change in the CeO_2_ reflections.

**Figure 3.**
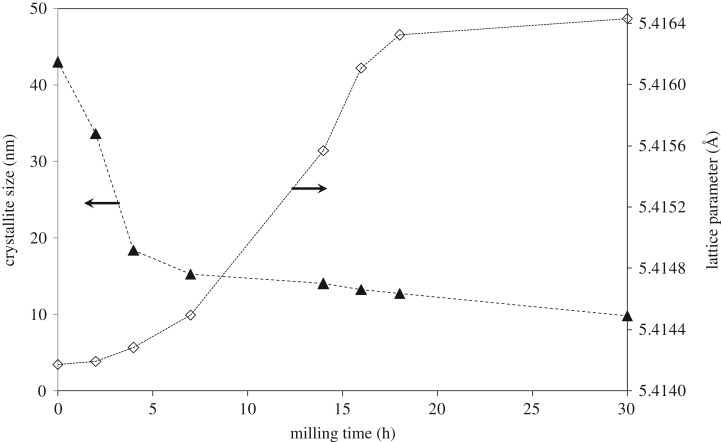
Crystallite size and lattice parameter of CeO_2_ in (CuO)_0.5_(CeO_2_)_0.5_ composite powder as a function of milling time.

The lattice parameter and the estimated crystallite size of the cubic CeO_2_ phase in the (CuO)_0.5_(CeO_2_)_0.5_ composite are shown in figure 3, as a function of milling time. The crystallite size is rapidly decreased during an early stage of the milling periods, less than approximately 5 h, followed by a gradual decrease up to 30 h milling. But the lattice parameter is only gradually increased during an early stage of milling, less than approximately 5 h, followed by a rather rapid increase with the increase of the milling time up to approximately 18 h, and then the variation becomes small up to 30 h milling. This indicates that although the change of the crystallite size is not directly related to the lattice parameter variation, the latter is activated only after the former event. This is consistent with the idea that the appearance of Cu and Cu_2_O phases is clearly observed when the milling duration is longer than 7 h, as shown in figure 1, because atomic-order mixing of powders that is prerequisite for the phase change is generally expected, particularly in the vicinity of the interphase interface, only after effective repeated folding of particles during milling, the so-called kneading effect, producing fine layered structures inside powder. It is also suggested that a steady state of the structural variation of CeO_2_ is attained after approximately 18 h milling. The decrease of crystallite size and the increase of lattice parameter of CeO_2_ with milling time are also reported [44–48].

The lattice parameters and crystallite sizes of the cubic CeO_2_ phase and the specific surface areas (BET) of 18 h-milled samples with various CuO contents are listed in table 1. With an increase of mol% CuO contents, the lattice parameter of CeO_2_ is increased, the average crystallite size is decreased and the BET surface area of CuO–CeO_2_ powder composite is decreased. Because the smaller crystallite size may cause an elongation of the cubic lattice in the nano-sized CeO_2_ phase [47], which is ascribed to the lattice strain from the formation of the Ce^3+^ cations and the corresponding oxygen vacancies, the observed tendency is consistent. The slight decrease of the BET area with the CuO ratio is probably due to formation of the soft Cu phase during 18 h milling (figure 2), which may cause agglomeration of powders.

**Table 1.** Lattice parameter, crystallite size, of cubic CeO2 and BET surface area for various (CuO)_x_(CeO2)_1-x_ samples after 18h milling

Sample	Lattice parametter (A^o^)	Crystallite size (nm) d[220]	S_A_ (BET) m^2^g^−1^
(CuO)_0.8_(CeO_2_)_0.2_	5.4168(6)	11.3	17.5–19.7
(CuO)_0.5_(CeO_2_)_0.5_	5.4163(2)	12.8	18.6–21.9
(CuO)_0.8_(CeO_2_)_0.2_	5.4160(0)	13.6	19.4–22.3
(CuO)_0.2_(CeO_2_)_0.8_	5.4158(9)	14.4	21.1–23.7
CeO_2_	5.4152(4)	15.6	23.3–25.6

### 3.2. Morphological features

**Figure 4.**
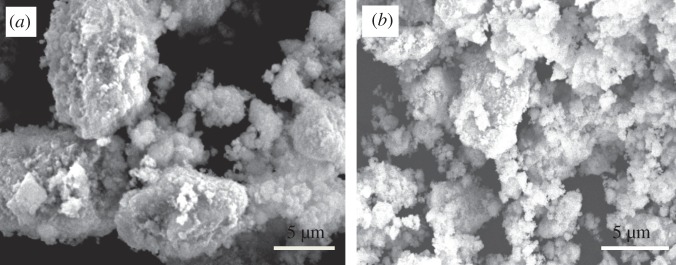
SEM micrograph of 18 h-milled powders; (*a*) (CuO)_0.8_(CeO_2_)_0.2_ and (*b*) (CuO)_0.2_(CeO_2_)_0.8._

**Figure 5.**
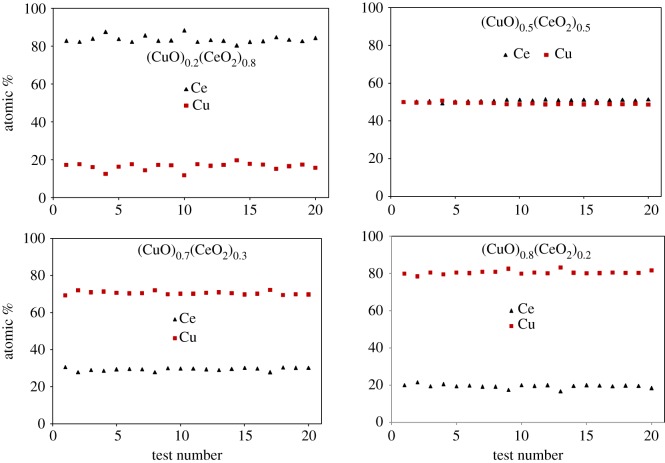
EDX analysis on various (CuO)_x_(CeO_2_)_1−x_ samples after 18 h milling; 15 kV.

The morphological features and the compositional homogeneity are studied by SEM and EDX. Similar morphology is observed for all the milled samples, where aggregations of packed particles from a few hundred nanometres to micrometres in size exist, as shown in figure 4. Some reports show that the CuO is incorporated and evenly distributed into the CeO_2_ structure [49,50]. As shown in figure 5, with the use of each beam size adjusted as 10 µm, the areal average concentrations of each 18 h-milled sample with a different overall composition (20, 50, 70 or 80 mol% CuO) exhibit high homogeneity in terms of Ce and Cu contents, judging from the EDX analyses at 20 randomly selected squares. The magnification of SEM is fixed at 220 times. This strongly suggests that the dimensional size of the Cu or Cu_2_O phase, as observed in figure 2, is much smaller than 10 µm. Contamination from the milled media (ZrO_2_ balls and a steel vial) is also examined by EDX, and the maximal contamination level is less than 1 wt% for Fe while no contamination is detectable for Cr, ZrO_2_, and so on, after 18 h milling.

### 3.3. Reduction behaviour by TPR

**Figure 6.**
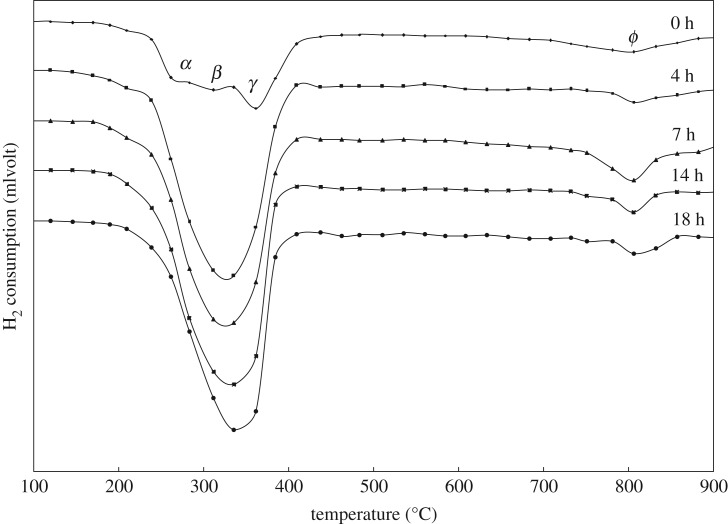
TPR of (CuO)_0.5_(CeO_2_)_0.5_ composite powder with milling time.

The H_2_-TPR profiles of the (CuO)_0.5_(CeO_2_)_0.5_ samples milled for 0, 4, 7, 14 and 18 h are shown in figure 6. For powders without milling (0 h milling), i.e. by mixing the oxide powders, the reduction of (CuO)_0.5_(CeO_2_)_0.5_ is characterized by rather combined three peaks (*α*, *β*, and *γ*) in the range of 220–420°C and one broad *ϕ* peak in the range of 700–880°C. After 4 h milling, the former peaks (*α*, *β* and *γ*) merge into one large broad peak cantered around 300°C–320°C with some skewed symmetry. The position and the intensity of the peak are not largely changed with further milling, but the skewness is somewhat increased, with the peak top shifting to the higher temperatures with milling time up to 18 h (figure 6). The progression of the milling process should produce a large amount of bounding interface/interphase between CuO and CeO_2_, and some Cu atoms would even be penetrated into CeO_2_, producing Ce_1−x_Cu_x_O_2_ solid solutions near the interface vicinity. These would facilitate the mobility of oxygen atoms for both phases, resulting in promoted valence variation of cations and leading to the observed large areas of H_2_ consumption peak. As for the latter peak, the broadness of the peak is slightly reduced with milling time, whereas the peak top is around 800°C.

**Figure 7.**
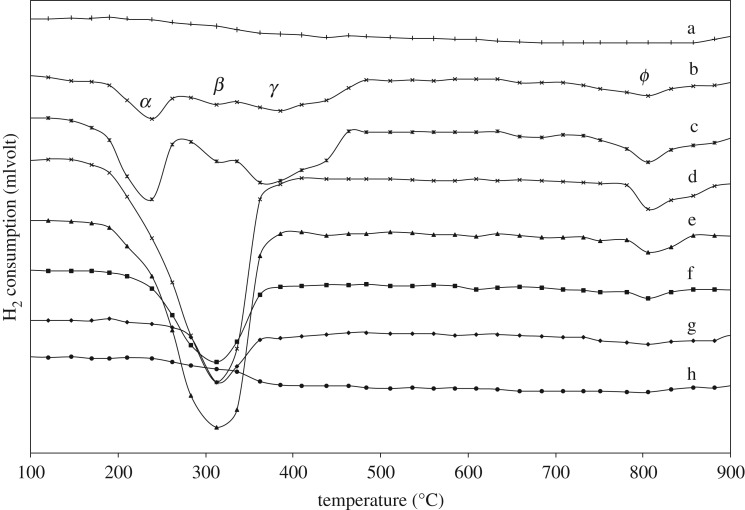
TPR of (a) CeO_2_, (b) CuO powder for 0 h milling and (CuO)_x_(CeO_2_)_1−x_ powder after 18 h milling (*x*= (c) 1, (d) 0.8, (e) 0.5, (f) 0.3, (g) 0.2, (h) 0).

Figure 7 shows the H_2_-TPR profiles of various (CuO)_x_(CeO_2_)_1−x_ powders (x = 1, 0.8, 0.5, 0.3, 0.2, 0) after 18 h milling, compared with pure CeO_2_ (a) and CuO (b) powders without milling (0 h milling). For pure CuO (x = 1), the reduction is characterized by rather combined three peaks (*α*, *β*, and *γ*) in the range of 200–470°C and one broad *ϕ* peak in the range of 750–850°C (figure 7*b,c*), regardless of milling or without milling, strongly indicating that the powder morphology and the milling-induced defects are not the major factors influencing the reduction behaviour. In addition, the range of reduction temperatures is wider compared with that of the (CuO)_0.5_(CeO_2_)_0.5_ composite (figure 6: 0 h), indicating multiple reduction steps are involved with possible inhomogeneous reactions. But, only two apparent peaks are observed for lower CuO contents (figure 7*d–g*), and the intensity of both peaks is reduced when CuO content is decreased, demonstrating the major contribution of CuO to the H_2_ consumption. For pure CeO_2_ (figure 7*a,h*), there is no distinguished H_2_-TPR peak in the temperature range observed, while the consumption of H_2_ steadily exists above 350°C up to approximately 900°C for reducing CeO_2_ to Ce_2_O_3_, where the reduction first occurs near the surface defects [51,52] followed by the formation of intermediate CeO2-x and complete transformation to Ce_2_O_3_.

It is reported by Fierro *et al*. [53] that the TPR characteristics could be affected by mass transfer limitations and experimental operating variables such as the initial amount of reducible species, the initial H_2_ concentration, the total gas flow rate, the heating rate, and the activation energy of the reaction. They claim that desorption of H2 attached to the reduced Cu metal surface could exhibit the apparent double-peak behaviour, which may be affected by water vapour produced by the reduction process, and that the H_2_-TPR profile of CuO depends on the particle size and surface area, where the peak top is higher by 288°C for particle sizes of 425 approximately 850 microns compared with less than 100 microns [53]. A similar tendency but with a much larger peak shift (over 373°C) is also reported by Luo *et al*. [49], who claim that the hydrogen spillover effect is the reason for the difference between CO-TPR and H_2_-TPR.

Kim *et al.* [54] report that there is an incubation period prior to reduction, which is longer at lower temperatures. The tendency is in agreement with the general theory of nucleation and growth, where the number of newly formed nuclei is copious at lower temperatures, but because the growth is very much limited at lower temperatures, the phase existence is not easily detected by x-ray diffractometry. They claim that CuO reduction is generally easier than Cu_2_O reduction with H_2_-TPR, with the apparent activation energy for Cu_2_O close to twice that of CuO, but when the H_2_ flow rate is not high enough for avoiding the rate-limiting of the reduction process, a sequential reduction process such as CuO → (Cu_4_O_3_→) Cu_2_O → Cu may happen. Our experimental condition is: 15^o^C min^−1^, 50 mg and 25 cc min^−1^ of 5%H_2_ flow. This is close to the condition for the appearance of the sequential process due to the ‘lean H_2_’ flow. The incubation period and the sequential/simultaneous reduction process are also reported by Kim *et al.* [54], where, with the lean H_2_ condition, the CuO, Cu_2_O and Cu are simultaneously observed after a certain incubation period according to the time-resolved XRD. This is consistent with our thought experiments (not shown here), where the 3 phases are simultaneously observed when the H2-TPR is stopped at 280°C and kept for 15 min, while only CuO is observed when the sample is immediately quenched from 280°C to RT.

Consequently, the *α* peak for pure CuO reduction, as shown in figure 7, should be mainly attributed to direct reduction of CuO particles into Cu in the surface vicinity, and some simultaneous reduction to Cu_2_O (or Cu_4_O_3_) should be involved with our experimental operating variables, somewhat contributing to the *α* peak. Because the rate of nucleation and growth of each reduced phase is different, which involves the shape of nuclei and the nucleation sites besides the spillover effect of H_2_ on the metallic Cu, one rate-determining step would gradually dominate the sequential CuO reduction to Cu_2_O and Cu, exhibiting the broad β peak for the slow reduction process, which is related to the larger activation energy for Cu_2_O formation compared with direct Cu formation. Before the end of the sequential reaction, some H_2_ molecules would be dissociated from H atoms on newly formed Cu on the powder surface, penetrating into the Cu(/Cu_2_O)/CuO particles, and at certain temperature(s), depending on the surrounding condition, the remaining CuO would be reduced to Cu either directly or sequentially through Cu_2_O (or Cu_4_O_3_), exhibiting the γ peak. The extended TPR peak profile as high as 470°C or even 480°C strongly indicates the difficulty of reduction due to longer the diffusion path for H atoms or H_2_ molecules as well as the escape of produced H_2_O molecules. It is acknowledged that the larger the CuO particles, the thicker the reduced Cu phase; this covers the particle and shows the bulk-like behaviour of CuO reduction. Thus, the *α* and *β* peaks are probably caused by surface-vicinity reduction with separate locations, whereas the γ peak would be due to reduction inside the particles. At the high temperature *ϕ* peak, it may cause the Cu surface to interact with the SiO_2_ quartz reactor [55,56].

The apparent increase of the H_2_-TPR starting temperature for the *α* peak, as shown in figure 6, compared with pure CuO in figure 7*b,c* is probably due to variation on the mass transfer limitations affected by experimental operating variables such as the initial amount of reducible species and effective total gas flow rate through mixing CeO_2_ particles with CuO particles. But, the end temperature for the *γ* peak, as shown in figure 6, is much decreased, compared with pure CuO shown in figure 7*b,c*. This must be the mixing effect of CeO_2_ particles with CuO particles, in that the contact point or the interphase interface plays an important role in the nucleation and growth of the reduced phase such as Cu, or even Cu_2_O. Owing to the valence change of cations requiring high electron mobility as well as the higher oxygen mobility near the contact point, the nucleation event is much more active in the vicinity, and with the fast surface diffusion of Cu atoms the nucleation is immediately followed by growth or accumulation of the new phase, always leaving some area of fresh CuO surface contacting with H_2_/Ar gas. In the reduction process, the diffusion paths for H atoms or H_2_ molecules as well as the escape path of the produced H_2_O molecules are established, and the reduction is continued until all the CuO is consumed.

There are many reports on the CuO–CeO_2_ powder systems and the reduction study. Many researchers report that ceria in the CuO–CeO_2_ powder promotes surface species reduction of highly dispersed copper oxide [57–64] and the CuO particles with smaller sizes are easier to reduce [65]. According to Liu *et al.* [58], the reduction peak at lower temperature is attributed to copper oxide clusters strongly interacting with ceria, whereas the peak at higher temperature is attributed to larger CuO particles, not associated with ceria. A similar tendency in the H_2_-TPR profile is also reported by Luo *et al.* [60] and Xiaoyuan *et al.* [61]. Furthermore, Tang *et al.* [66] report that the lower temperature peak is assigned to reduction of amorphous CuO clusters closely interacting with the CeO_2_, while Luo *et al.* [48] claim that with increasing CuO content three peaks appear, with the lowest temperature deriving from reduction of finely dispersed CuO, the intermediate temperature associated with reduction of Cu^2+^ ions in Cu_x_Ce_1−x_O_2−δ_ solid solutions, and the additional highest one due to reduction of bulk CuO.

As observed in figure 6, with increasing milling time on (CuO)_0.5_(CeO_2_)_0.5_ composite samples, the skewness of H_2_-TPR profile is somewhat increased with the peak top shifting to the higher temperatures. Also, in figure 7, as the CuO content is decreased in various 18 h-milled (CuO)_x_(CeO_2_)_1−x_ powders, the H_2_ absorption at lower temperatures involved with the *α* peak is gradually diminished, with the peak tops of the main H_2_-TPR profiles around 310–330°C, which is similar to that shown in figure 6. Wang *et al.* claim that [49] Cu atoms embedded in ceria have an oxidation state higher than those of the cations in Cu_2_O or CuO, where (i) Cu in the doped sample is only partially reduced to metallic Cu, especially at low temperatures as CuO → Cu_2_O → Cu, because Cu embedded in ceria is difficult to reduce, (ii) The lattice of the Ce_1−x_Cu_x_O_2_ systems (fluorite-type) is highly distorted with local order defects and multiple cation-oxygen distances, and (iii) Doping CeO_2_ with Cu introduces large strain and O vacancies.

This is consistent with our results in that, at the early stage of milling such as before 7 h milling, the ultrafine mixture of CuO/CeO_2_ is more effective for H_2_-TPR profile, which corresponds to the rapid crystallite size reduction before 4 h milling, as shown in figure 3. Here, the large area of CuO/CeO_2_ interphase interface is extremely efficient for producing the path for H_2_ diffusion and H_2_O escape, besides the effective valence change of cations, where the x-ray diffractometry in figure 1 identifies only CuO and CeO_2_ phases. But, the longer the milling time, the more intense the formation of Ce_1−x_Cu_x_O_2_ solid solution systems, where the higher peak temperature for the H_2_-TPR profile should be observed due to Cu embedded in ceria as milling proceeds. The results are also consistent with our x-ray results shown in figure 1, where atomic-order mixing is achieved at least near the interface vicinity of CuO/CeO_2_, producing Cu and Cu_2_O after 7 h milling, and that the clear increase in the lattice parameter is observed (figure 3) after approximately 7 h milling.

It is thus quite reasonable to consider that, at the early stage of milling, the major H_2_-TPR peaks caused by milling of CuO/CeO_2_ are attributed to reduction of Cu^2+^ ions in the interfacial vicinity of CeO_2_, which exist in the ultrafine mixture of CuO/CeO_2_ or some Cu_2_O/CeO_2_. The CuO particles are highly dispersed, producing many active sites for Cu^2+^ reduction on the interphase interface with CeO_2_. With the increase of milling time, some Cu atoms are even incorporated into CeO_2_, causing formation of Ce_1−x_Cu_x_O_2_ solid solutions. The large intensity and the broad profile of the H_2_-TPR peaks would be observed due to the dual effect.

**Figure 8.**
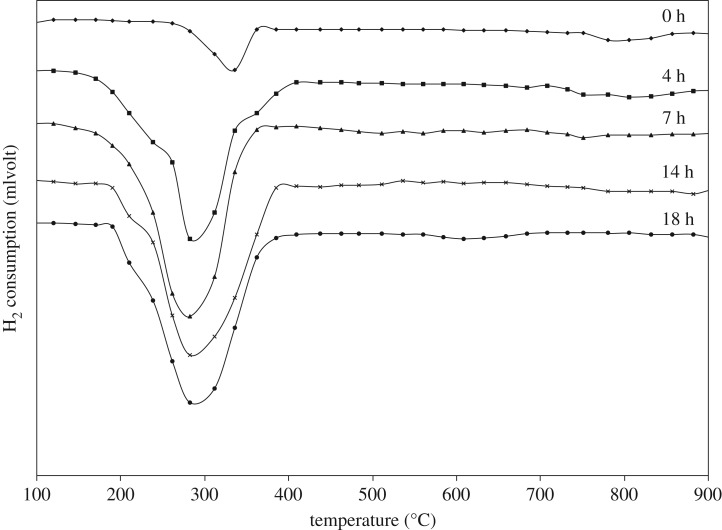
TPR of (CuO)_0.5_(CeO_2_)_0.5_ powder with milling time after the first TPR cycle.

Figure 8 shows the repeated H_2_-TPR cycles of milled (CuO)_0.5_(CeO_2_)_0.5_ composites (50 mol% CuO milled for 0, 4, 7, 14 and 18 h) in figure 6, each after the first TPR. The peak intensity for the 0 h milled sample is largely decreased, when compared with the first run, with high temperature peak(s) remaining, suggesting agglomeration/recrystallization of CuO particles that produce bulk-like CuO after heating to 900°C. When increasing the milling time, the CeO_2_ particles are finely distributed into the CuO particles, or some Ce–Cu–O solid solutions are produced, which prevents agglomeration/recrystallization of CuO, leading to the slow decrease observed in the peak intensity. The temperatures of the reduction start as well as the peak top of the H_2_ consumption are lowered compared with the first run. On considering that the pure CuO is reduced in the wide range of temperatures (200–470°C, figure 7*b,c*) regardless of milling or without milling under this particular experimental condition, some CuO is heterogeneously reduced (lean H_2_-TPR) at low temperatures (200–250°C) to produce Cu and Cu_x_O simultaneously. But, because the (CuO)_0.5_(CeO_2_)_0.5_ composite powders are finely mixed with milling, even after the H_2_-TPR to 900°C, the elemental mixture is maintained, and the CuO reduction is activated by surface contact with CeO_2_.

**Figure 9.**
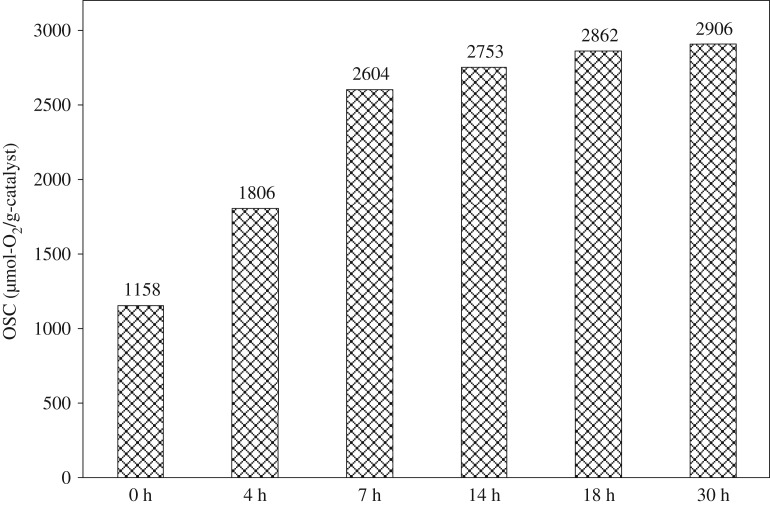
Total OSC at 300°C of (CuO)_0.5_(CeO_2_)_0.5_ powder with milling time.

Figure 9 shows the total OSC at 300°C of various milled samples (50 mol% of CuO) after 0, 4, 7, 14, 18 and 30 h milling. The total OSC is increased with milling time, with the rapid increase from 0 to 7 h milling followed by the gradual increase from 7 h to 30 h. This is consistent with figure 1 (x-ray results) and figure 3 in that the rapid atomic-order mixing is effective during the early stage milling of 7 h, evidenced by both solid state reactions and crystallite size variations. It is also noted that, because the lattice parameters are largely varied after 7 h milling, the CuO/CeO_2_ interphase interface creation and the corresponding atomic contact would be more important for the effective OSC than formation of the Ce–Cu–O solid solution, although both contribute to the OSC promotion. That is, the activated valence change of Cu^2+^/Cu^+^/Cu assisted by neighbouring Ce-O bonds is extremely important, and the oxygen transport path is also critical for the OSC. The enhanced oxygen storage/transport would thus be realized through easy valence change of Cu^2+^/Cu^+^/Cu neighboured by Ce-O bonds and the large surface area with the large number of the available oxygen vacancies.

**Figure 10.**
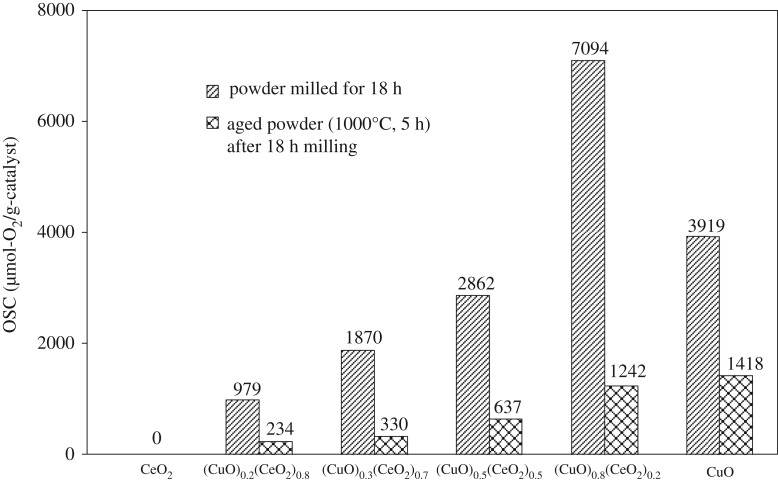
Total OSC at 300°C of milled samples for 18 h and after ageing at 1000°C for 5 h.

The total OSC at 300°C for the samples milled for 18 h is increased, as shown in figure 10, from 0 to 7094 µmol-O_2_/g-catalyst with increased CuO content from 0 to 80 mol%, but is decreased to 3919 µmol-O_2_/g-catalyst for pure CuO (BET surface area of 15–16 m^2^ g^−1^). After ambient ageing at 1000°C for 5 h, however, the total OSCs at 300°C are significantly reduced (figure 10), probably ascribed to the agglomeration/recrystallization of fine particles.

**Figure 11.**
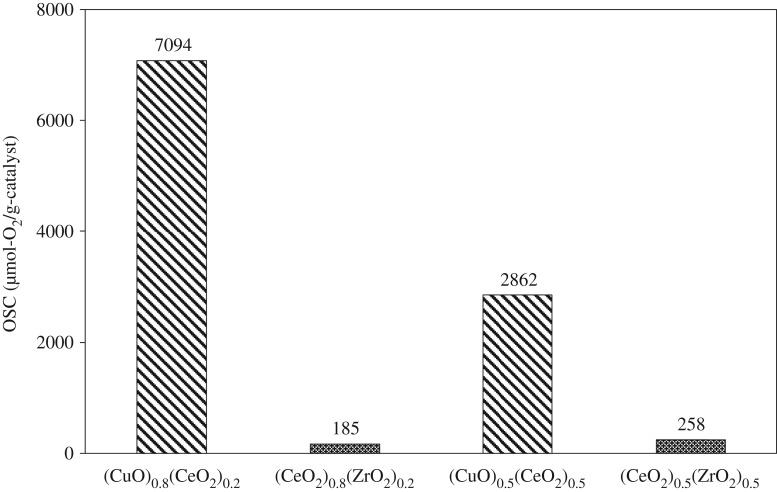
Comparison of total OSC at 300°C of CuO–CeO_2_ and CeO_2_–ZrO_2_-milled powder.

As shown in figure 11, the total OSCs of the CeO_2_-20 at %ZrO_2_ and the CeO_2_-50 at %ZrO_2_ at 300°C are 185 and 258 µmol-O_2_/g-catalysts, respectively, while those of the CuO–CeO_2_ catalyst system prepared with the same condition are at least one order greater. It clearly shows that the mechanically driven CuO/CeO_2_ system exhibits the high OSC property, and this should contribute to improving the performance of TWCs at lower temperatures.

## 4. Conclusion

Mechanical milling was applied to the CuO–CeO_2_ powder system to produce mixed-oxide catalysts. The milled sample was characterized by the use of XRD, SEM, TG-DTA, GC-TCD and BET analyses. Milling of powder mixtures of CuO and CeO_2_ showed the reduction of CuO when milling was processed in air. The crystallite size of ceria was rapidly decreased at the early stage of milling, followed by an increase of the lattice parameter, indicating formation of Ce_1−x_Cu_x_O_2_ solid solutions after the rapid crystallite size reduction. The redox property of milled CuO–CeO_2_ samples was investigated by H_2_-TPR. Three reduction peaks were observed for 0 h milling and only one broad peak for various milling times, where the valence change of Cu ions enhanced the redox activity. The higher OSC for the CuO–CeO_2_ system was observed with increased milling time. The total OSC of the CuO–CeO_2_ catalyst was much higher than that of the CeO_2_–ZrO_2_ traditional catalyst system at low temperature.

## Supplementary Material

XPS data

Reviewer comments

## Data accessibility

The datasets supporting this article have been uploaded as part of the electronic supplementary material.
